# A Case of Bronchopneumonia Presenting With Persistent Hiccups Successfully Treated With Clonazepam and a Traditional Kampo Medicine

**DOI:** 10.7759/cureus.75237

**Published:** 2024-12-06

**Authors:** Norihito Yoshida, Tatsuki Tanaka, Yusuke Suzuki, Ryogo Ohashi, Mai Hitaka, Shingo Ishii, Keisuke Yamazaki, Yasushi Ohashi

**Affiliations:** 1 Division of Nephrology, Toho University Sakura Medical Center, Sakura, JPN; 2 Division of Pulmonology, Toho University Sakura Medical Center, Sakura, JPN

**Keywords:** acute pneumonitis, chlorpromazine, hemodialysis, hiccups treatment, kampo medicine (japanese herbal medicine)

## Abstract

Persistent hiccups are rare but can serve as an early symptom of underlying conditions, including pulmonary infections and cerebrovascular disorders. This case highlights hiccups as a presenting symptom of bronchopneumonia in a hemodialysis patient and explores the effective use of chlorpromazine and Hange-koboku-to (HKT) as symptomatic therapies. Given the potential association of hiccups with neurological conditions, this case underscores the need for comprehensive diagnostic evaluation.

A 62-year-old man undergoing maintenance hemodialysis for end-stage renal disease presented with persistent hiccups lasting one week, accompanied by reduced oral intake. Imaging revealed ground-glass opacities in the right lower lobe and minimal bilateral pleural effusion. Sputum culture confirmed *Klebsiella* species, consistent with bronchopneumonia. Treatment included ampicillin/sulbactam (ABPC/SBT), azithromycin (AZM), chlorpromazine (37.5 mg/day, three times daily (t.i.d.)), and HKT (7.5 g/day, t.i.d.). Hiccups resolved within two days of initiating therapy, and both symptomatic treatments were discontinued by the fifth hospital day. At a two-week follow-up, the patient remained symptom-free with improved quality of life (QOL).

This case demonstrates persistent hiccups as a potential early symptom of bronchopneumonia, likely caused by diaphragmatic irritation. Non-pharmacological interventions failed to provide relief, but a combination of chlorpromazine and HKT effectively alleviated symptoms. HKT, a traditional Kampo medicine, may modulate neurotransmitter pathways and serve as an adjunctive treatment for refractory hiccups. Furthermore, persistent hiccups in hemodialysis patients warrant careful evaluation to exclude central nervous system (CNS) disorders, including brainstem infarctions. These findings underscore the importance of recognizing atypical presentations of pneumonia and tailoring a multimodal therapeutic approach. Persistent hiccups require careful evaluation to rule out cerebrovascular events, but in this case, they were caused by bronchopneumonia. This report highlights hiccups as an early symptom of bronchopneumonia and demonstrates the efficacy of chlorpromazine and HKT as symptomatic treatments. Recognizing atypical presentations of respiratory infections and adopting targeted therapies are essential for effective management.

## Introduction

Persistent hiccups, defined as episodes lasting over 48 hours, can significantly impair quality of life (QOL). These involuntary diaphragmatic contractions are mediated by a reflex arc involving the medulla oblongata and cervical (C3-C5) and thoracic (T6-T12) spinal segments [1,2]. While often triggered by common factors such as gastrointestinal or pulmonary irritants, persistent hiccups may indicate underlying conditions, including central nervous system (CNS) disorders, infections, metabolic imbalances, or cerebrovascular events [3]. Notably, hiccups have been reported as an early symptom of brainstem or medullary infarctions, highlighting their diagnostic importance in acute neurological conditions. In hemodialysis patients, persistent hiccups are rare but pose unique challenges due to their potential impact on nutritional intake and overall health. This report describes a case of persistent hiccups associated with bronchopneumonia in a hemodialysis patient, emphasizing the diagnostic approach and therapeutic management.

## Case presentation

A 62-year-old man presented to our hospital with persistent hiccups lasting one week. He reported a decrease in oral intake due to the hiccups. He denied abdominal pain, shortness of breath on exertion, weight gain, or prodromal symptoms of upper respiratory infection prior to the onset of hiccups. Despite attempting maneuvers such as breath-holding, the hiccups persisted. His medical history included end-stage renal disease secondary to type 2 diabetes mellitus, managed with maintenance hemodialysis three times per week, and gastroesophageal reflux disease (GERD). He had no history of smoking or alcohol use, no recent relocation, and no exposure to new pets. The patient was unemployed and had no known allergies.

On admission, the patient’s vital signs were as follows: height, 172 cm; weight, 65.5 kg (body mass index (BMI): 22.1 kg/m²); temperature, 36.2°C; blood pressure, 132/65 mmHg; pulse rate, 92 beats per minute (regular); respiratory rate, 20 breaths per minute; and oxygen saturation (SpO₂), 98% on room air.

On physical examination, conjunctivae appeared normal with no evidence of pallor. There was no cervical lymphadenopathy. Cardiac auscultation revealed regular heart sounds without murmurs or additional sounds. Lung auscultation detected wet rales localized to the right lower lung field. The abdomen was soft, non-distended, and non-tender. No peripheral edema was observed. Persistent hiccups were noted at intervals of 10-15 seconds during the examination. Additionally, a prominent superficial brachial artery was observed in the right upper arm. Chest radiography revealed ground-glass opacities localized to the right lower lung field, with no evidence of cardiomegaly. Electrocardiography demonstrated sinus rhythm at a rate of 91 beats per minute, without any significant ST-T wave abnormalities. Transthoracic echocardiography showed a preserved left ventricular ejection fraction of 64%, with no regional wall motion abnormalities. The inferior vena cava diameter was measured at 9 mm during expiration and 3 mm during inspiration, findings consistent with normal intravascular volume status. Blood tests revealed elevated white blood cell (WBC) count and C-reactive protein (CRP) levels, consistent with an inflammatory response. Given that the tests were performed post-dialysis, serum creatinine (Cr) and blood urea nitrogen (BUN) levels were low, and no electrolyte imbalances were observed. Liver function tests, creatine kinase-MB (CK-MB), and troponin I (Trop-I) levels were within normal limits, ruling out hepatic dysfunction and myocardial injury. There were no findings suggestive of thyroid or adrenal insufficiency. Additionally, tuberculosis-specific interferon-gamma release assay (T-SPOT), cytomegalovirus (CMV) antigen, β-D-glucan, SARS-CoV-2 PCR, and *Mycobacterium avium* complex (MAC) antibody tests were all negative, excluding tuberculosis, CMV infection, fungal infections, and COVID-19. Screening for autoimmune diseases yielded negative results, and Krebs von den Lungen-6 (KL-6) levels were normal, indicating no evidence of interstitial lung disease (Table 1).

**Table 1:** Laboratory findings Hb: hemoglobin, WBC: white blood cell count, Plt: platelet count, TP: total protein, Alb: albumin, AST: aspartate aminotransferase, ALT: alanine aminotransferase, ALP: alkaline phosphatase, γ-GTP: gamma-glutamyl transpeptidase, T-Bil: total bilirubin, CK: creatine kinase, CK-MB: creatine kinase-MB fraction, BUN: blood urea nitrogen, Cr: creatinine, eGFR: estimated glomerular filtration rate, CRP: C-reactive protein, Trop-I: troponin I, BNP: brain natriuretic peptide, ACTH: adrenocorticotropic hormone, TSH: thyroid-stimulating hormone, FT3: free triiodothyronine, FT4: free thyroxine, β-D-glucan: beta-D-glucan, T-SPOT: tuberculosis-specific interferon-gamma release assay, CMV antigen: cytomegalovirus antigen, SARS-CoV-2 PCR: severe acute respiratory syndrome coronavirus 2 polymerase chain reaction, RF: rheumatoid factor, IgG: immunoglobulin G, IgA: immunoglobulin A, IgM: immunoglobulin M, C3: complement component 3, C4: complement component 4, KL-6: Krebs von den Lungen-6, CH50: total complement activity, ANA: antinuclear antibody, anti-dsDNA IgG: anti-double-stranded DNA immunoglobulin G, anti-ssDNA IgG: anti-single-stranded DNA immunoglobulin G, MPO-ANCA: myeloperoxidase anti-neutrophil cytoplasmic antibodies, PR3-ANCA: proteinase 3 anti-neutrophil cytoplasmic antibodies, anti-MAC antibody: anti-mycobacterium avium complex antibody, NGSP: National Glycohemoglobin Standardization Program

Parameter	Result and unit	Reference range
WBC	10,360/μL	4,000-9,000/μL
Hb	11.3 g/dL	13.0-17.0 g/dL
Plt	212 × 10³/μL	150-400 × 10³/μL
TP	6.5 g/dL	6.5-8.3 g/dL
Alb	3.3 g/dL	3.5-5.0 g/dL
AST	19 IU/L	13-33 IU/L
ALT	8 IU/L	10-42 IU/L
ALP	59 U/L	38-126 U/L
γ-GTP	6 IU/L	8-61 IU/L
T-Bil	0.7 mg/dL	0.2-1.2 mg/dL
CK	282 U/L	60-287 U/L
CK-MB	13 IU/L	<24 IU/L
BUN	13.0 mg/dL	8-22 mg/dL
Cr	4.27 mg/dL	0.6-1.2 mg/dL
eGFR	12 mL/minute/1.73m²	>60 mL/minute/1.73m²
Uric acid	2.0 mg/dL	3.6-7.2 mg/dL
Glucose	109 mg/dL	70-99 mg/dL
HbA1c	5.2% (NGSP)	4.0%-5.6%
Na	139 mEq/L	135-145 mEq/L
K	3.5 mEq/L	3.5-5.0 mEq/L
Cl	101 mEq/L	98-106 mEq/L
Ca	9.5 mg/dL	8.4-10.2 mg/dL
P	2.7 mg/dL	2.5-4.5 mg/dL
CRP	6.75 mg/dL	<0.3 mg/dL
Trop-I	Negative	Negative
BNP	632 pg/mL	<100 pg/mL
ACTH	37.5 pg/mL	7.2-63.3 pg/mL
Cortisol	12.1 ng/mL	6.2-19.4 ng/mL
TSH	2.69 µIU/mL	0.27-4.20 µIU/mL
FT3	2.20 pg/mL	2.3-4.1 pg/mL
FT4	1.40 ng/dL	0.9-1.7 ng/dL
β-D-glucan	22 pg/mL	<60 pg/mL
T-SPOT	Negative	Negative
CMV antigen	Negative	Negative
SARS-CoV-2 PCR	Negative	Negative
RF	<3 IU/mL	<15 IU/mL
IgG	877 mg/dL	700-1,600 mg/dL
IgA	167 mg/dL	70-400 mg/dL
IgM	21 mg/dL	40-230 mg/dL
C3	118 mg/dL	79-152 mg/dL
C4	29 mg/dL	16-38 mg/dL
KL-6	208 U/mL	<500 U/mL
CH50	43.7 IU/mL	30-50 IU/mL
ANA	<40 titer	<40 (speckled)
Anti-dsDNA IgG	3.4 IU/mL	<7 IU/mL
Anti-ssDNA IgG	<1.0 IU/mL	<1.0 IU/mL
MPO-ANCA	Negative	Negative
PR3-ANCA	Negative	Negative
Anti-MAC antibody	Negative	Negative

**Figure 1:**
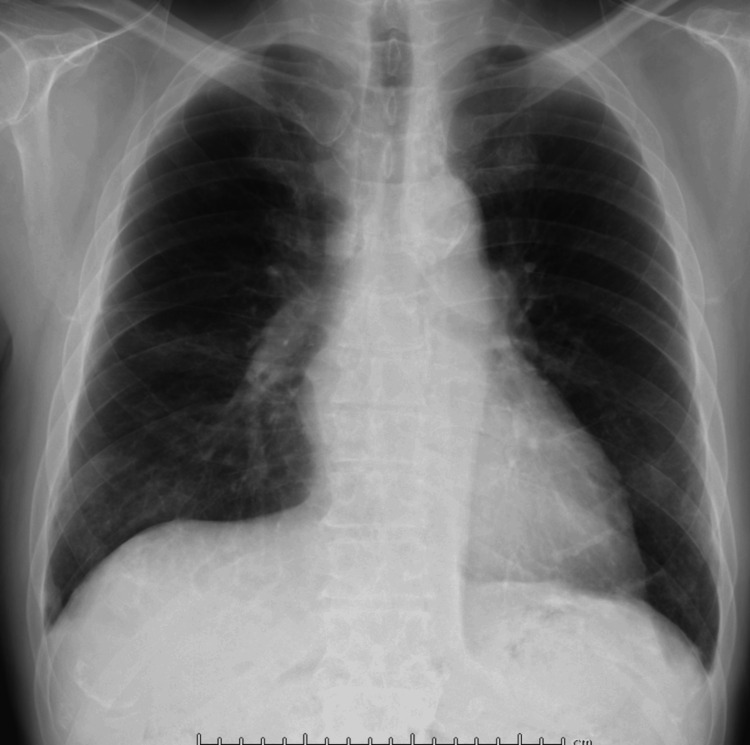
Chest radiograph A chest radiograph demonstrating ground-glass opacities in the right lower lung field. No evidence of cardiomegaly is observed.

**Figure 2:**
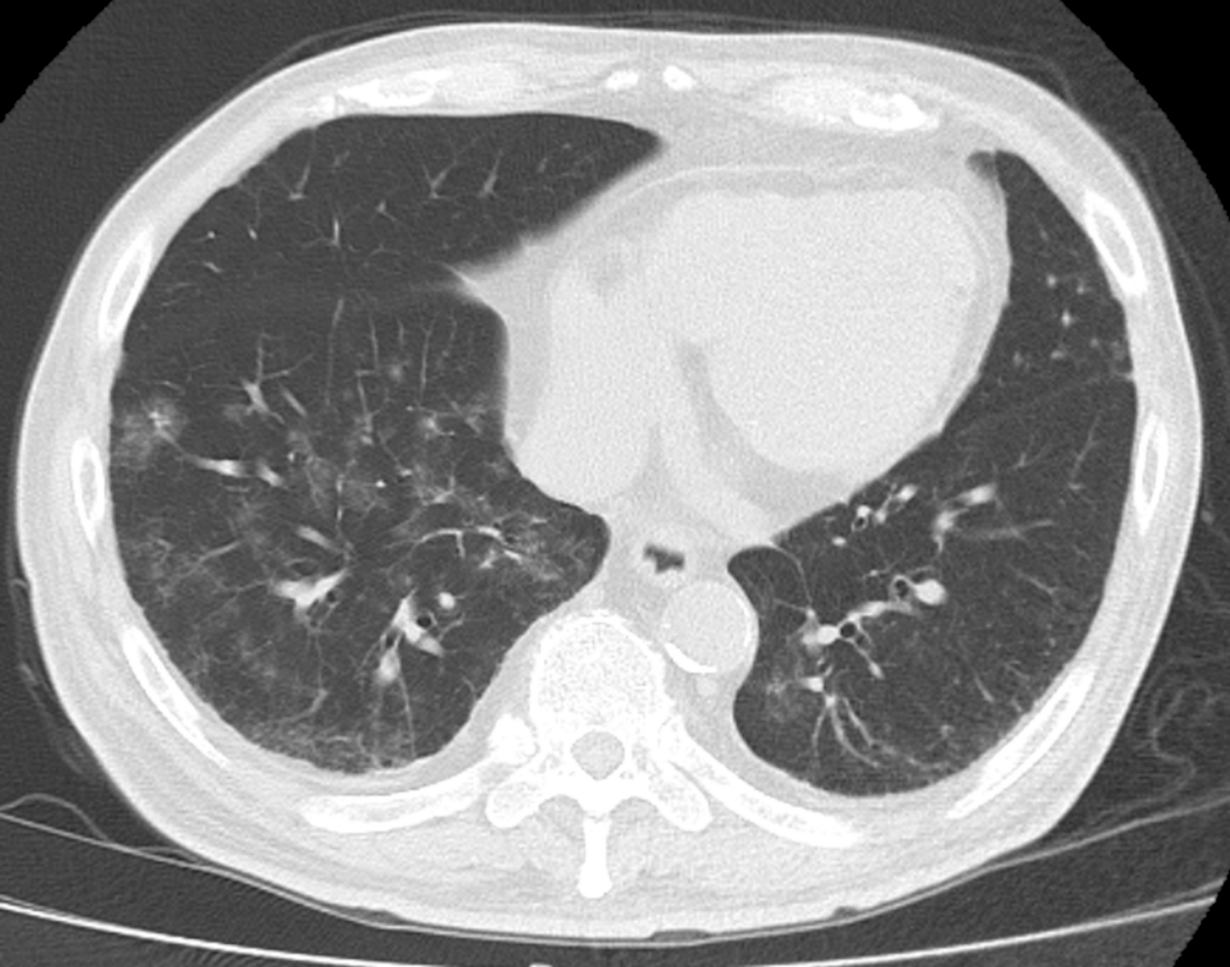
Pre-treatment non-contrast CT scan of the chest A non-contrast CT scan of the chest showing ground-glass opacities along the airways of the right lower lobe. Minimal bilateral pleural effusion is noted, with no evidence of mediastinal lymphadenopathy. CT: computed tomography

**Figure 3:**
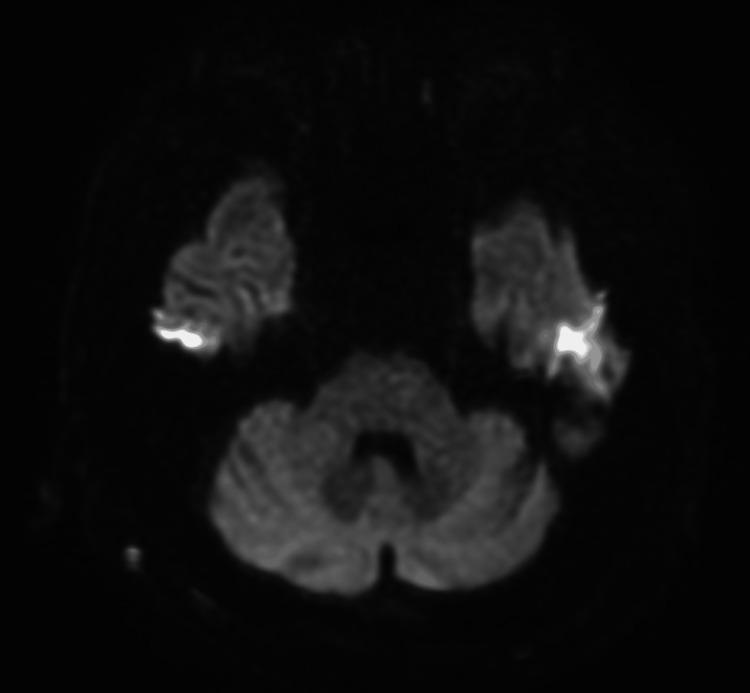
Brain MRI (DWI of the brain) DWI of the brain reveals no acute stroke findings in the medulla or cerebellum. MRI: magnetic resonance imaging, DWI: diffusion-weighted imaging

**Figure 4:**
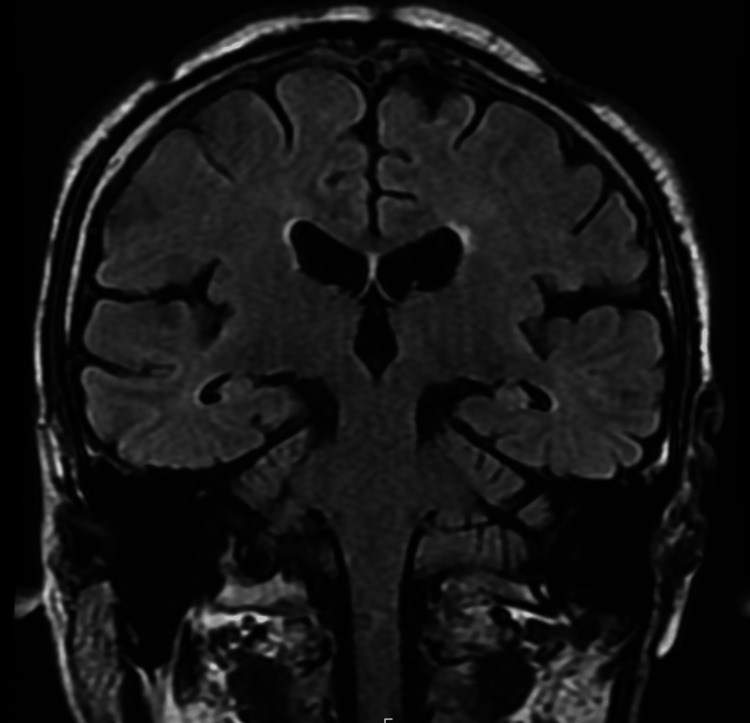
Brain MRI (FLAIR imaging) FLAIR imaging shows no evidence of chronic ischemic changes in the brainstem. MRI: magnetic resonance imaging, FLAIR: fluid-attenuated inversion recovery

A chest radiograph showed ground-glass opacities in the right lower lung field, with no evidence of cardiomegaly (Figure 1). Non-contrast computed tomography (CT) imaging from the neck to the pelvis revealed ground-glass opacities in the right lower lobe and minimal bilateral pleural effusion. There was no evidence of pulmonary infiltrates, nodular lesions, or mediastinal lymphadenopathy. Additionally, no abnormalities were identified in the neck, such as tumors, nor were there findings of gastrointestinal dilation, ascites, or pelvic masses (Figure 2). Diffusion-weighted imaging (DWI) on brain magnetic resonance imaging (MRI) revealed no acute cerebrovascular lesions in the medulla (Figure 3). Magnetic resonance angiography (MRA) showed no evidence of cerebral aneurysms. Additionally, fluid-attenuated inversion recovery (FLAIR) imaging on brain MRI demonstrated no chronic ischemic changes or signs of prior infarctions in the medulla (Figure 4).

The patient was diagnosed with bronchopneumonia, with consideration given to atypical pneumonia, and was treated with antibiotics (ampicillin/sulbactam (ABPC/SBT) and azithromycin (AZM)) alongside symptomatic therapy consisting of chlorpromazine and Hange-koboku-to (HKT). Chlorpromazine was initiated at a total daily dose of 37.5 mg, administered as 12.5 mg, three times daily (t.i.d.), while HKT, also known as Banxia Houpu Tang, was prescribed at a total daily dose of 7.5 g, divided into three doses (2.5 g, t.i.d.). Within approximately three hours of initiating treatment, the frequency of hiccups significantly decreased, enabling the patient to resume oral intake. By the second hospital day, the hiccups had nearly resolved.

On the fifth hospital day, chlorpromazine and HKT were discontinued. Antibiotic therapy was completed with a five-day course of ABPC/SBT and a three-day course of AZM. Given the presence of minimal bilateral pleural effusion observed on chest CT, the target dry weight (DW) during dialysis sessions was adjusted downward by 1 kg. Due to the atypical nature of the pneumonia findings, a consultation with the respiratory medicine department was sought, and a bronchoscopy was performed.

**Figure 5:**
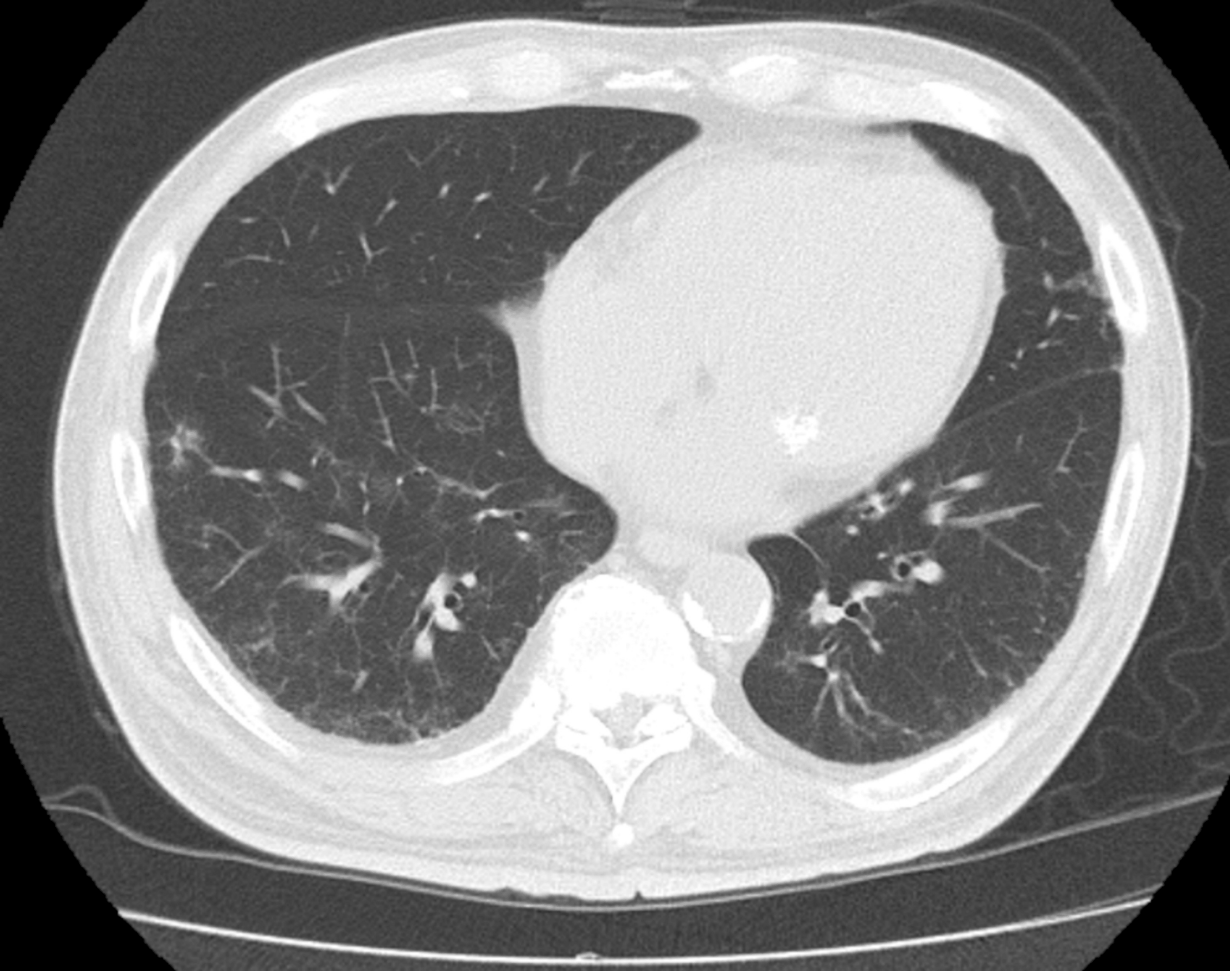
Post-treatment chest CT scan Ground-glass opacities show improvement. Bilateral pleural effusions persist. CT: computed tomography

Following discontinuation of antibiotics, chlorpromazine, and HKT, the patient experienced no recurrence of hiccups, and oral intake remained stable. However, there was a decline in activities of daily living (ADL) due to hospitalization, necessitating rehabilitation. The patient was discharged home on the 11th hospital day. Follow-up chest CT imaging, performed two weeks after discharge, showed improvement in ground-glass opacities, although small bilateral pleural effusions persisted (Figure 5). The patient reported no recurrence of symptoms and a return to normal daily activities, confirming a favorable post-treatment course.

## Discussion

Reports of persistent hiccups associated with bronchopneumonia in hemodialysis patients are exceedingly rare. The reflex arc responsible for hiccups, first described in 1943, involves afferent, central, and efferent pathways, including the medulla oblongata, cervical spinal cord (C3-C5), and thoracic spinal cord (T6-T12). This model remains the basis for understanding the neural mechanisms underlying hiccups [1,2]. Although the precise pathophysiology is not fully understood, hiccups are believed to arise from myoclonic contractions of the diaphragm and intercostal muscles triggered by disruptions in these neural circuits.

Common triggers for hiccups include gastric distension from aerophagia, overeating, or consumption of carbonated beverages, as well as spicy foods, alcohol, smoking, and gastrointestinal or pulmonary irritants. Persistent hiccups are frequently linked to central nervous system (CNS) conditions such as cerebrovascular accidents and brain tumors [3]. Peripheral causes often include gastroesophageal reflux disease (GERD), hiatal hernia, myocardial ischemia, and aortic aneurysms. Additional etiologies, including otolaryngological conditions such as pharyngeal diseases or metabolic disturbances such as uremia and electrolyte imbalances, may　also contribute [4].

A thorough diagnostic approach is critical and should encompass a detailed history (including medical, surgical, and medication histories, as well as dietary, smoking, and alcohol use patterns), comprehensive physical examination, and targeted imaging studies of the head, chest, and abdomen. Upper gastrointestinal endoscopy is reserved for cases where other findings are inconclusive [4].

The management of persistent hiccups prioritizes addressing the underlying etiology. For idiopathic or refractory cases, symptomatic treatments may be employed. Physical maneuvers such as breath-holding, swallowing, deep breathing, and the Valsalva maneuver are commonly utilized to mitigate acute episodes [4,5]. Among these, breath-holding may reduce hiccup frequency by increasing arterial pCO₂ levels, thereby influencing diaphragmatic excitability [6].

Pharmacological interventions become necessary when non-pharmacological measures are insufficient. Proton pump inhibitors (PPIs) are effective for GERD-related hiccups, while dopamine receptor antagonists (e.g., chlorpromazine) and GABA receptor agonists (e.g., baclofen) are widely used for persistent symptoms. Chlorpromazine, introduced in the 1950s, remains the standard pharmacological treatment for intractable hiccups, although alternatives such as metoclopramide are available [6,7].

HKT, a traditional Japanese Kampo medicine derived from classical Chinese medicine, has shown promise as an adjunctive treatment for persistent hiccups. This formulation consists of five herbal ingredients: Pinelliae Tuber (Hange), derived from the tuber of *Pinellia ternata*; Magnoliae Cortex (Koboku), the bark of *Magnolia officinalis*; Perillae Folium (Soyo), the leaves of *Perilla frutescens*; Zingiberis Rhizoma (Shokyo), the rhizome of *Zingiber officinale*; and Glycyrrhizae Radix (Kanzo), the root of *Glycyrrhiza glabra*. Together, these components are believed to contribute to the regulation of gastrointestinal and respiratory symptoms.

HKT has been shown to modulate neurotransmitter pathways, including serotonin, norepinephrine, dopamine, and substance P. It may also improve swallowing reflexes and exhibit antidepressant effects [8,9]. Unlike chlorpromazine, which directly antagonizes dopamine receptors to alleviate diaphragmatic spasms, HKT likely exerts its effects by balancing brain monoamine systems. This complementary mechanism may enhance the efficacy of symptom management in hiccup cases where standard therapies fall short [10].

In the present case, persistent hiccups were attributed to bronchopneumonia based on imaging and microbiological findings. Sputum cultures identified *Klebsiella* species, supporting the diagnosis. Cardiovascular and CNS causes, including brainstem infarction, were excluded through electrocardiography, cardiac ultrasound, and brain MRI. GERD was deemed unlikely given the patient’s PPI therapy and lack of supporting clinical symptoms. COVID-19, another potential cause, was excluded through negative testing [11].

Bronchopneumonia typically warrants a five- to seven-day course of antibiotics; however, given the patient’s immunocompromised status as a hemodialysis recipient, an extended treatment duration was implemented [12]. The hiccups associated with the right lower lobe pneumonia in this case were likely attributable to diaphragmatic irritation, a recognized mechanism in similar presentation [13]. Sputum cultures identifying *Klebsiella* species supported the appropriateness of the chosen antibiotic regimen. Furthermore, alternative etiologies, including tuberculosis and collagen vascular diseases, were excluded through negative results for antinuclear antibody (ANA), anti-neutrophil cytoplasmic antibody (ANCA), hypoglobulinemia, β-D-glucan, and T-SPOT testing. Additionally, the absence of *Mycobacterium avium* complex (MAC) antibodies further ruled out non-tuberculous mycobacterial infections. Findings from bronchoalveolar lavage ruled out malignancy or other significant abnormalities, reinforcing the diagnosis of bacterial pneumonia. Uremia-related hiccups were also considered unlikely, as dialysis adequacy was confirmed with no reported interruptions [14]. Although bilateral pleural effusion persisted, it was considered that further downward adjustments to the DW during dialysis might still be possible. However, the effusion was unlikely to be the primary cause of the patient’s symptoms in this case.

This case underscores the need for thorough diagnostic evaluation and tailored management strategies in hemodialysis patients presenting with persistent hiccups. While it was not possible to definitively determine whether symptom resolution resulted from the antibiotic therapy or adjunctive treatments, the multimodal approach employed was likely instrumental in achieving clinical improvement. The absence of symptom recurrence after pneumonia resolution further suggests that GERD was not a primary contributing factor. Future studies are warranted to elucidate the underlying mechanisms of persistent hiccups and explore novel therapeutic options beyond empirical treatments such as chlorpromazine.

## Conclusions

This case highlights the importance of comprehensive evaluation and individualized treatment for persistent hiccups, particularly in hemodialysis patients. Successful symptom resolution was achieved through a multimodal approach, addressing both the underlying bronchopneumonia with antibiotics and symptomatic management using chlorpromazine and HKT. The absence of recurrence after discontinuing therapy suggests the effectiveness of this strategy. Future research is needed to develop evidence-based treatments for persistent hiccups in high-risk populations.
